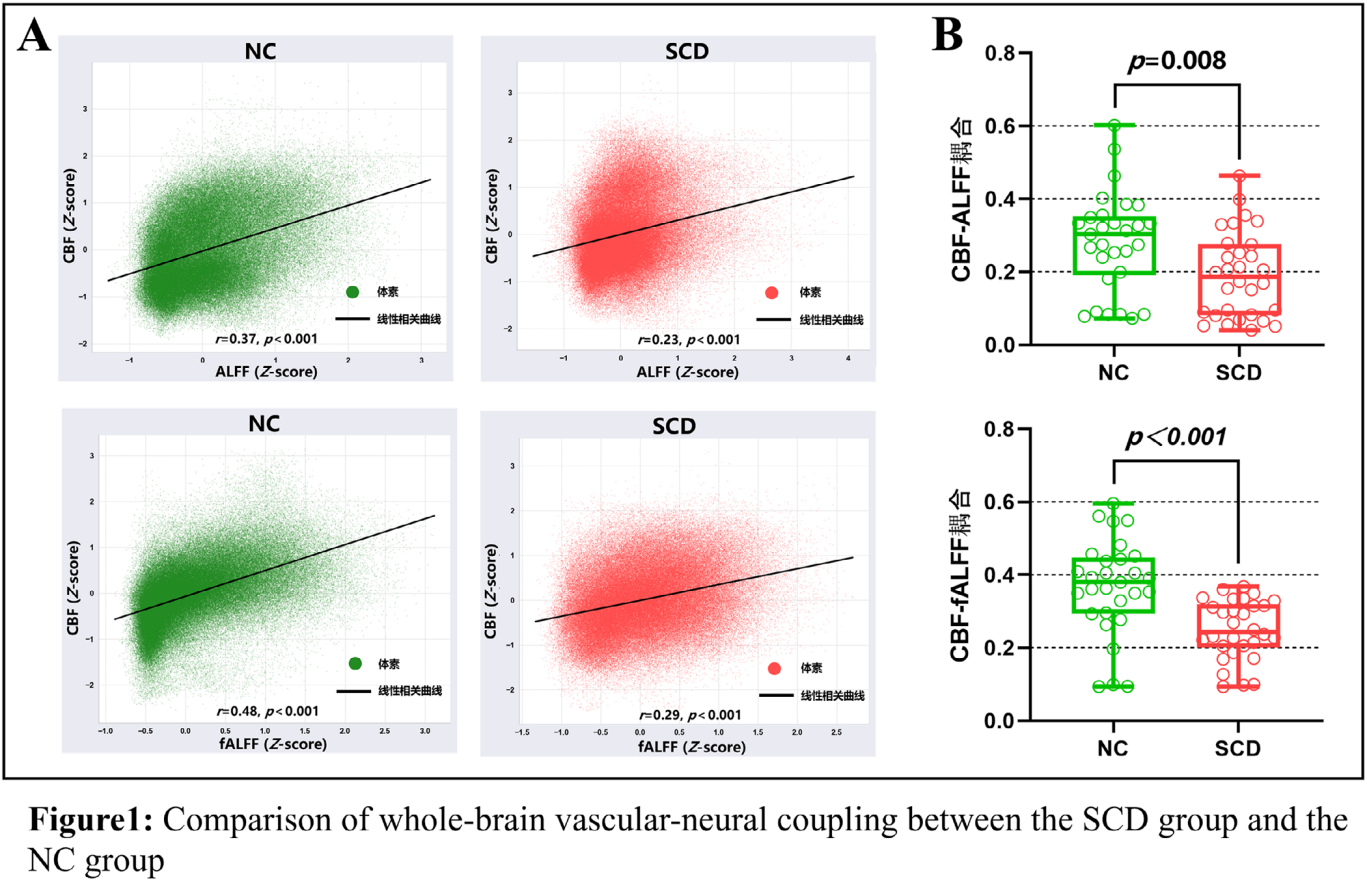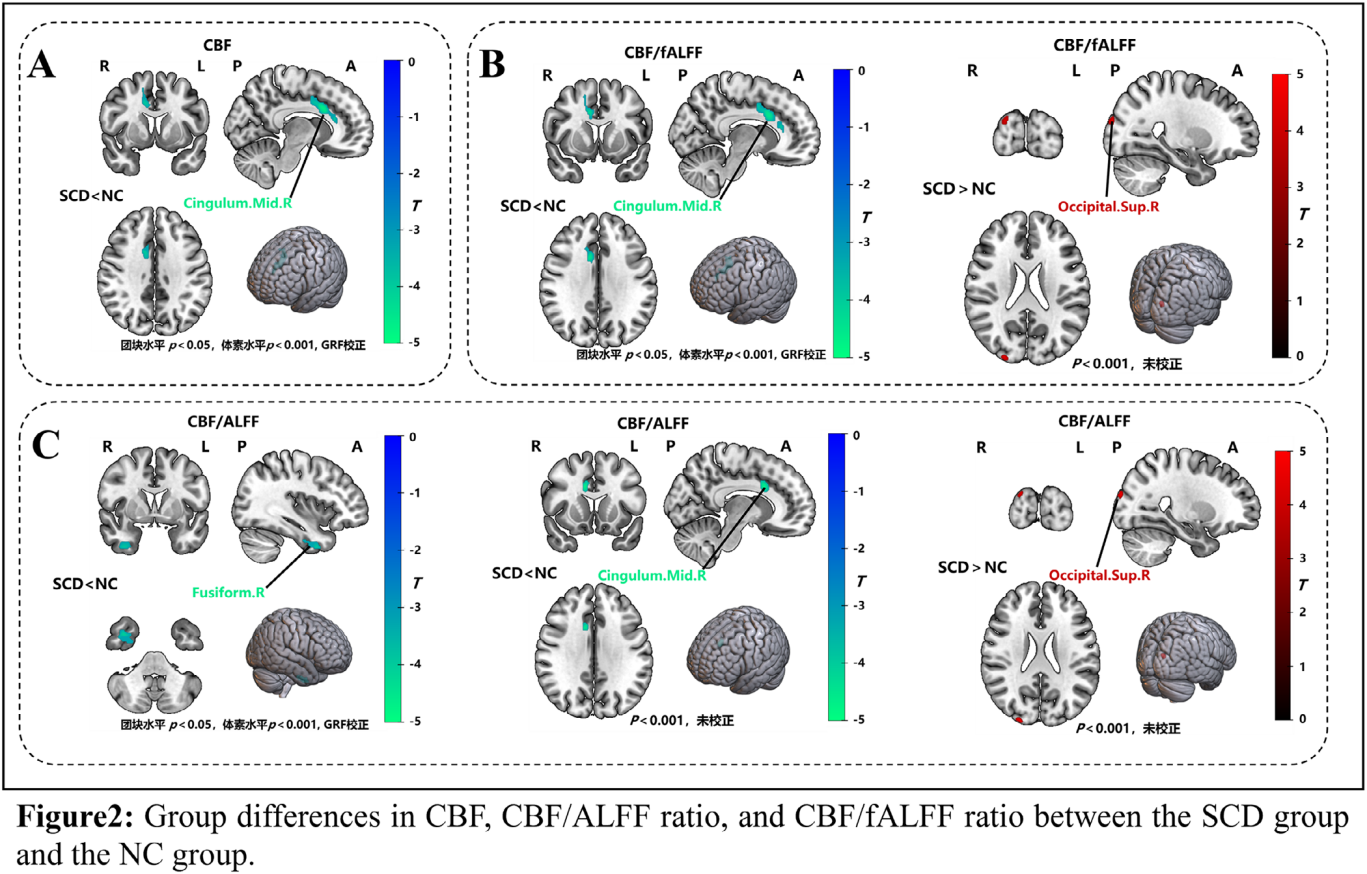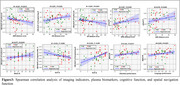# Association between Neurovascular Coupling Changes and Spatial Navigation Impairment in Individuals with Subjective Cognitive Decline: A 5.0T MRI Study

**DOI:** 10.1002/alz70856_098846

**Published:** 2025-12-24

**Authors:** Xuefeng Ma, Bing Zhang, Futao Chen, Xiang Fan

**Affiliations:** ^1^ Department of Radiology, Nanjing Drum Tower Hospital, Affiliated Hospital of Medical School, Nanjing University, Nanjing, Jiangsu, China; ^2^ Peking University Shenzhen Hospital, Shenzhen, Guangdong, China

## Abstract

**Background:**

Subjective cognitive decline (SCD) is a high‐risk population for preclinical Alzheimer's disease (AD). Neurovascular coupling (NVC) reflects the complex physiological processes of neuronal activity and cerebral blood flow (CBF), providing new mechanistic insights into neurodegenerative diseases. Spatial navigation impairment is a sensitive biomarker at the SCD stage. Ultra‐high field magnetic resonance imaging (UHF‐MRI) has unique advantages in exploring the neural mechanisms of neurodegenerative diseases. However, changes in NVC in the SCD based on high‐resolution imaging with UHF‐MRI and their relationship with spatial navigation function remain unknown. To explore changes in NVC and their relationship with spatial navigation function in the SCD population based on 5.0T MRI arterial spin labeling (ASL) and multi‐echo functional magnetic resonance imaging.

**Method:**

30 normal controls (NC) and 29 SCD individuals were recruited for neuropsychological assessments, spatial navigation tests, plasma biomarker detection, and 5.0T MultiEcho fMRI and ASL scans. Amplitude of Low Frequency Fluctuation(ALFF), fractional ALFF (fALFF) were calculated through fMRI, and CBF was calculated through ASL. Changes in whole gray matter CBF and ALFF, CBF and fALFF coupling between the two groups, and each voxel's CBF/ALFF, CBF/fALFF ratio were compared. Spearman correlation analysis assessed the relationship between CBF/ALFF, CBF/fALFF ratios, plasma biomarkers, and spatial navigation function, cognitive performance. Classification efficiency of cognitive scale scores, spatial navigation distance error, CBF/ALFF, CBF/fALFF ratios for SCD and NC was evaluated.

**Result:**

The SCD population showed reduced NVC and greater distance errors in AN and EN. The SCD group had significantly lower CBF and CBF/ALFF, CBF/fALFF ratios in specific brain regions compared to the NC group. Correlation analysis showed significant correlations between CBF/ALFF, CBF/fALFF ratios, plasma biomarkers, and spatial navigation function, cognitive performance. ROC curve analysis showed the combined model of brain imaging indicators and spatial navigation distance error had the highest classification efficiency for SCD and NC.

**Conclusion:**

This study indicates that the SCD population exhibits changes in NVC and spatial navigation impairment, with a significant association between the two. Brain NVC decoupling may be the neuropathological mechanism of spatial navigation impairment in the SCD population.